# Biotechnical paving of recombinant enterocin A as the candidate of anti-*Listeria* agent

**DOI:** 10.1186/s12866-014-0220-8

**Published:** 2014-08-28

**Authors:** Xiaoyuan Hu, Ruoyu Mao, Yong Zhang, Da Teng, Xiumin Wang, Di Xi, Jianzhong Huang, Jianhua Wang

**Affiliations:** Key Laboratory of Feed Biotechnology, Ministry of Agriculture, Beijing, 100081 China; Gene Engineering Laboratory, Feed Research Institute, Chinese Academy of Agricultural Sciences, 12 Zhongguancun Nandajie St., Haidian District, Beijing, 100081 P. R. China; College of Life Science, Engineering Research Center of Industrial Microbiology, Fujian Normal University, Fuzhou, 350108 China

**Keywords:** Enterocin A, *Listeria ivanovii*, Antimicrobial activity, *Pichia pastoris*, Expression

## Abstract

**Background:**

Enterocin A is a classic IIa bacteriocin isolated firstly from *Enterococcus faecium* CTC492 with selective antimicrobial activity against *Listeria* strains. However, the application of enterocin A as an anti-*Listeria* agent has been limited due to its very low native yield. The present work describes high production of enterocin A through codon optimization strategy and its character study.

**Results:**

The gene sequence of enterocin A was optimized based on preferential codon usage in *Pichia pastoris* to increase its expression efficiency. The highest anti-*Listeria* activity reached 51,200 AU/ml from 180 mg/l of total protein after 24 h of induction in a 5-L fermenter. Recombinant enterocin A (rEntA), purified by gel filtration chromatography, showed very strong activity against *Listeria ivanovii* ATCC 19119 with a low MIC of 20 ng/ml. In addition, the rEntA killed over 99% of tested *L. ivanovii* ATCC19119 within 4 h when exposed to 4 × MIC (80 ng/ml). Moreover, it showed high stability under a wide pH range (2–10) and maintained full activity after 1 h of treatment at 80°C within a pH range of 2–8. Its antimicrobial activity was enhanced at 25 and 50 mM NaCl, while 100–400 mM NaCl had little effect on the bactericidal ability of rEntA.

**Conclusion:**

The EntA was successfully expressed in *P. pastoris*, and this feasible system could pave the pre-industrial technological path of rEntA as a competent candidate as an anti-*Listeria* agent. Furthermore, it showed high stability under wide ranges of conditions, which could be potential as the new candidate of anti-*Listeria* agent.

## Background

Bacteriocins are antimicrobial peptides synthesized in the ribosome and secreted into medium to establish a competitive advantage in their environment by eliminating competitors to gain resources [1]. Bacteriocins are generally classified in terms of size, structure, and modifications. Class I bacteriocins are lantibiotics. Class II bacteriocins consist of small peptides that do not contain modified residues. Class III bacteriocins usually are large and heat-labile proteins [2]. The well-known bacteriocin is nisin, a class I bacteriocin, which is widely used in commerce [3]. Recently, many reports clearly indicate that bacteriocins of class IIa have greater potential as antimicrobial agents [4] with a narrower inhibitory spectrum to *Listeria* strains than nisin [5]. *Listeria*, the most common pathogen in food, can lead the host to suffer from serious diseases such as enteritis, sepsis, meningitis and abortion [6]. The mortality rate caused by listeriosis is between 15 and 30% [7,8]. Additionally, some strains of *L. monocytogenes* easily acquire resistance to many antibiotics [9]. To control food contamination and listeriosis effectively, more or better anti-listerial drugs are needed.

Enterocin A (EntA), with many antimicrobial merits, is a class IIa bacteriocin that was first isolated from *Enterococcus faecium* CTC492 in the mid-1990s. Its mature form is composed of 47 amino acids with two disulfide bridges [10]. It shows high activity, particularly against *Listeria* species at nanomolar concentrations [11]*.* The native EntA has proven to effectively inhibit *L. monocytogenes* in fermented foods [12,13]. However, the low levels of bacteriocins secreted from natural strains do not meet the requirements of the industrial scale and have limited its application to study stages thus far. Therefore, various heterologous expressions were attempted in lactic acid bacteria, *Escherichia. coli (E.coli)* and yeast [12,14–16], but their actual production levels were not desirable and left room for improvement. *Pichia pastoris* is considered to be a promising system because the target protein can be directly secreted into culture medium. It was reported that the production and bactericidal titer of enterocin P expressed by *P. pastoris* X-33 was 3.7- and 16-fold higher (28.2 μg/ml and 1,024 BU/ml), respectively, than that from the native *E. faecium* P13 [17]; in fact, even though the level of 45.1 μg/ml of recombinant enterocin A expressed by *P. pastoris* [18] was still too low for its industrial production and end application, it demonstrates the potential to increase its productivity to be as high as possible and to further easily characterize its purification and properties. However, there are only few studies at the modification of bacteriocin genes, such as gene synthesis or codon optimization, which is considered as a promising technique for increasing protein expression level [19]; thus, further work with this system is necessary to achieve an increased protein expression level of target gene.

Due to the high anti-*Lister* activity of EntA and its low yield either in native strain and recombinant expression system, the EntA gene was optimized by the preferential codon usage of *P. pastoris* and was expressed into medium as recombinant EntA (rEntA). The purification of rEntA from ferment supernatant was tried by four methods including gel filtration chromatography, then the antimicrobial activity, proteolytic sensibility and stabilities of heat, pH and salt of purified rEntA were examined.

## Results

### Construction and transformation of the expression vector

**Figure 1:**
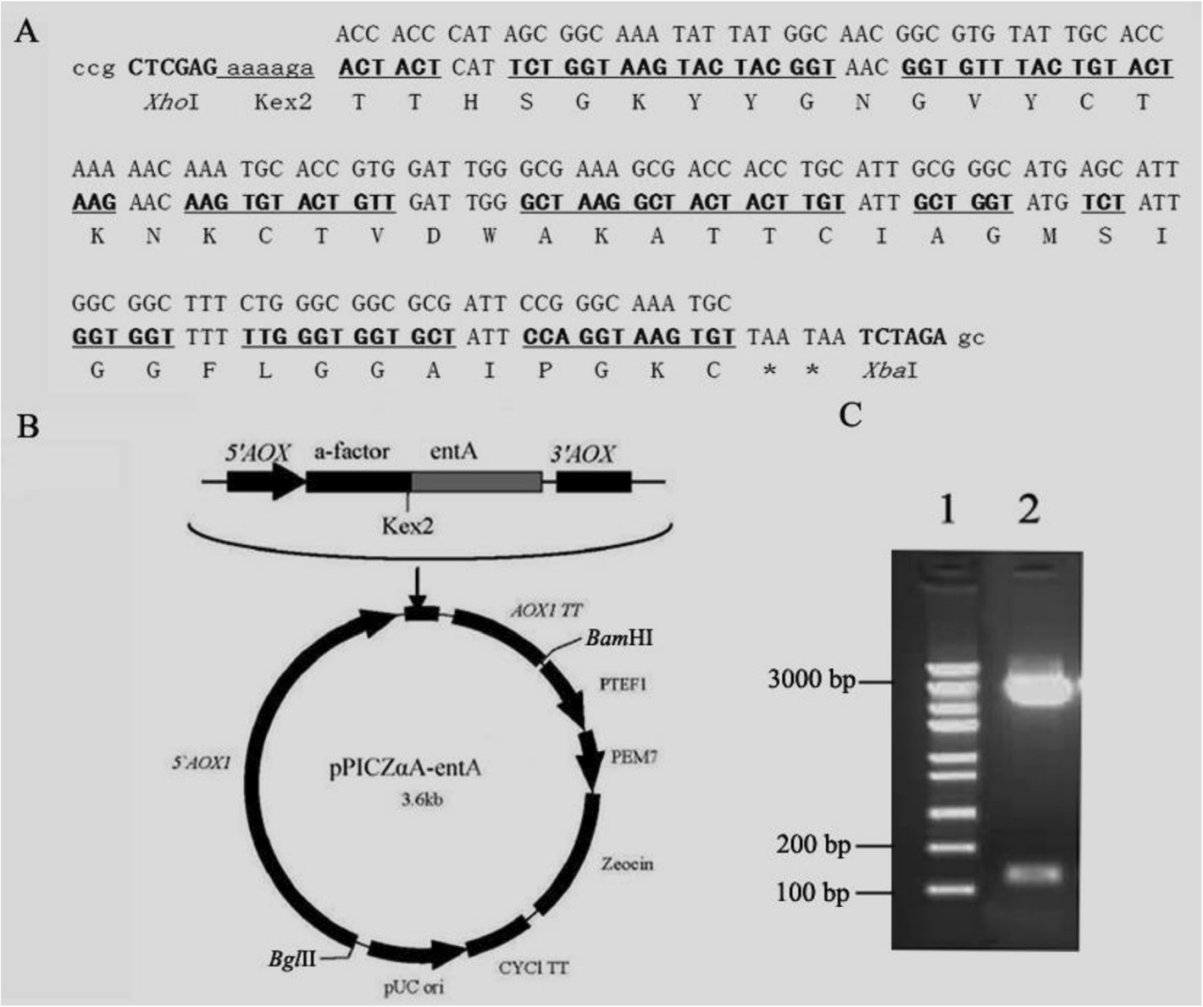
**Construction of the expression plasmid pPICZ** **α** **A-EntA. A**, The nucleotide sequence of EntA and its corresponding amino acid sequence. The upper line indicates the wild-type EntA gene sequence. The middle line is the codon-optimized EntA gene sequence. Optimized codons are underlined with boldface type. The lower line represents the amino acid sequence of EntA. The termination codon is marked by an asterisk. **B**, Map of the recombinant plasmid pPICZαA-EntA. **C**, Electrophoretic analysis of the recombinant vector containing the EntA gene. Lane 1, DNA marker; lane 2, pPICZαA-EntA digested by *Xho*I and *Xba*I.

Compared to naturally occurring EntA, the base codons coding for 37 residues (78.72%) in total 47 amino acids were optimized by the preferential codon usage of *P. pastoris* (Figure 1A). The GC content of the full target sequence increased from 41.13% to 41.9%. The gene sequence of the optimized EntA was synthesized and inserted into pPICZαA between *Xho*I and *Xba*I sites (Figure 1B, C). The expression vector pPICZαA-EntA was transferred into competent *E. coli* DH5α cells. Resulting transformants were confirmed by PCR and DNA sequencing. Correct plasmid and control vector pPICZαA were linearized by *Pme*I and transferred into competent *P. pastoris* X-33 cells by electroporation. Positive transformations were screened and confirmed by colony PCR.

### Expression of rEntA in shake flasks and at the fermenter level

**Figure 2:**
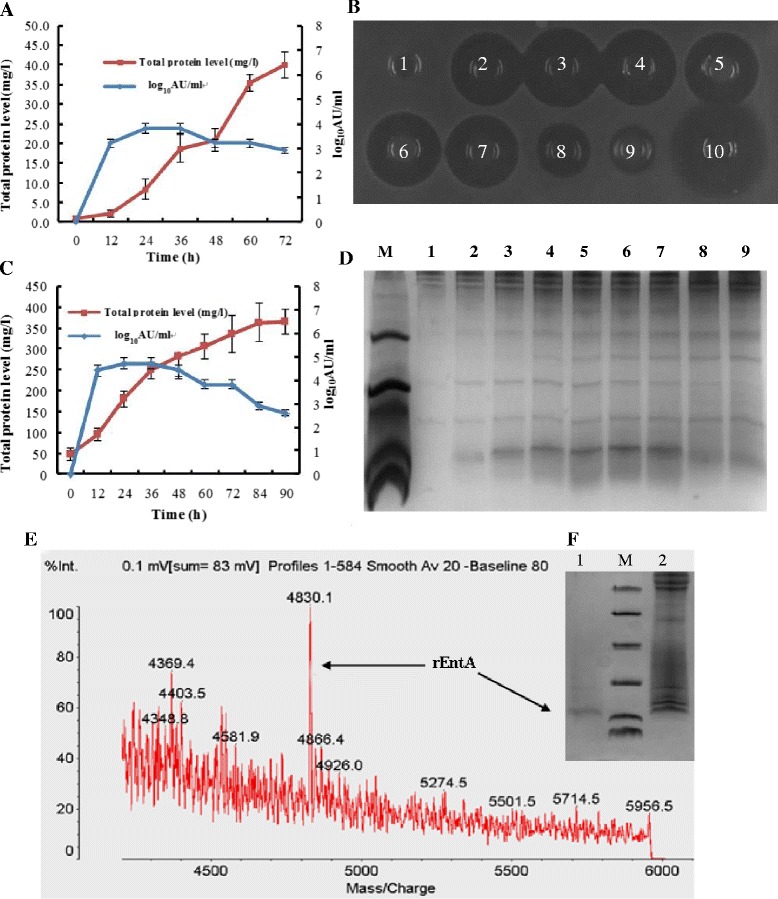
**Expression and purification of rEntA. A**, Total secreted protein level and antimicrobial titer of the fermentation supernatants of recombinant *P. pastoris* at the shake-flask level (bars represent the standard error of the mean). **B**, Antimicrobial activity of the fermentation supernatants of recombinant *P. pastoris* at the fermenter level. 1–9, 50 μl supernatant taken at 0, 12, 24, 36, 48, 60, 72, 84, and 90 h of induction, respectively; 10, 1 μg ampicillin. **C**, The total secreted protein level and antimicrobial titer in the fermenter level (bars represent the standard error of the mean). **D**, Tricine-SDS-PAGE analysis of rEntA secreted in the fermentation supernatant of *P. pastoris* cultures at the fermenter level. Lane M, 5 μl molecular mass standards (from top to bottom: 40, 25, 15, 10, 4.6 and 1.7 kDa); Lanes 1–9, 20 μl supernatant taken at 0, 12, 24, 36, 48, 60, 72, 84 and 90 h of induction, respectively. **E**, MALDI-TOF map of rEntA. **F**, Purification and identification of rEntA. Lane 1, purified rEntA (0.1 μg); Lane M, 5 μl molecular mass standards (from top to bottom: 40, 25, 15, 10, 4.6 and 1.7 kDa). Lane 2, 10 μl of rEntA supernatant taken at 24 h of induction.

The heterologous expression of rEntA in *P. pastoris* X-33 was induced by methanol at the concentration of 0.5% and analyzed by agar diffusion and Tricine-SDS-PAGE. *P. pastoris* X-33 containing the empty pPICZαA vector was used as a negative control. As shown in Figure 2A, after 12 h of methanol induction, the antibacterial activity of the supernatants of *P. pastoris* X-33 (pPICZαA-EntA) was observed. Its antibacterial activity reached maximum with 6,400 AU/ml after 24 h of methanol induction. However, the antimicrobial activity decreased from 48 to 72 h. No antibacterial activity was detected in the supernatants of *P. pastoris* X-33 (pPICZαA). The results of the MALDI-TOF MS for fermentation supernatants indicated that the molecular weight of rEntA was 4,830.1 Da, which was consistent with its theoretical value of 4,829 Da (Figure 2E).

To increase the production of rEntA, high-density fermentation of the recombinant yeast was performed using a 5-L fermenter. Although the total supernatant protein and biomass reached 365 mg/l and 343 g/l after induction for 90 h, the maximal antimicrobial activity was 51200 AU/ml (180 mg/l) after induction for 24 h (Figure 2C), which was 8-fold higher than that found at the shake-flask level. Figures 2B and D clearly showed that rEntA was rapidly degraded after 72 h of induction. Moreover, the expression of rEntA in the fermenter could be detected directly by Coomassie blue staining (Figure 2D), while its expression in the shake-flask could only be detected by silver staining (data not shown).

### Purification of rEntA

The rEntA was purified from the ferment supernatant after a 24-h induction in a 5-L fermenter. The bacteriocin activity of 6.40 × 10^5^ AU/mg with a 2.25-fold increase was obtained after gel filtration. The purified rEntA was analyzed by Tricine-SDS–PAGE and showed a band at 4.8 kDa representing the target protein band (Figure 2F), corresponding with its theoretical molecular weight.

### Antimicrobial spectrum of rEntA

Only *L. ivanovii* ATCC19119*, E. faecalis* CGMCC1.130 and *E. faecalis* CGMCC1.2024 were sensitive to rEntA in the 16 tested strains. Other Gram-positive bacteria, such as *E. faecium* CGMCC1.2136, *S. aureus* ATCC25923, *S. epidermidis* ATCC26069, *B. licheniformis* CGMCC1.265, and *B. coagulans* CGMCC1.2407, were found to be resistant to rEntA. All of the Gram-negative bacteria strains were resistant to rEntA in this assay (Table 1). The MIC and MBC of rEntA against *L. ivanovii* ATCC19119 were 20 ng/ml and 80 ng/ml, respectively, and were lower than those of ampicillin (390 ng/ml and 1560 ng/ml, respectively).

**Table 1:** **Antimicrobial spectrum of rEntA**

**Strains**	**Antimicrobial activity**
Gram-positive	
*Listeria ivanovii* ATCC19119	**+**
*Enterococcus faecium* CGMCC1.2136	**-**
*Enterococcus faecalis* CGMCC1.130	**+**
*Enterococcus faecalis* CGMCC1.2024	**+**
*Staphylococcus aureus* ATCC 25923	**-**
*Staphylococcus epidermidis* ATCC26069	**-**
*Bacillus licheniformis* CGMCC1.265	**-**
*Bacillus coagulans* CGMCC1.2407	**-**
*Bacillus subtilis* ATCC6633	**-**
*Lactococcus lactis* (Stored in our lab)	**-**
*Bifidobacterium bifidum* CGMCC1.2212	**-**
Gram-negative	**-**
*E. coli* ER2566	**-**
*E. coli* CVCC 195	**-**
*E. coli* CMCC 44102	**-**
*Pseudomonas aeruginosa* CVCC 2087	**-**
*Salmonella enteritidis* CVCC3377	**-**

Note: “+” refers to positive antimicrobial activity (inhibition zone > 6 mm); “**-**” refers to negative antimicrobial activity (inhibition zone ≤ 6 mm).

### In-vitro killing curve assay

**Figure 3:**
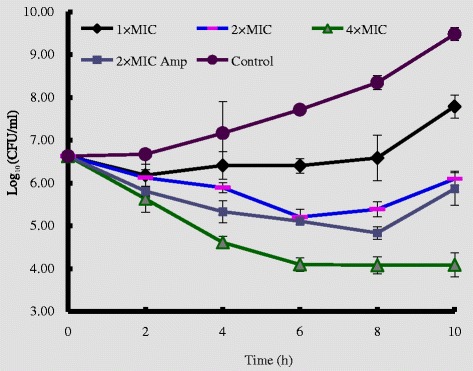
**Time-kill curves of rEntA.** *L. ivanovii* ATCC19119 was incubated in the presence of medium alone or in the presence of 1×, 2×, or 4× MIC of rEntA. Ampicillin of 2 × MIC was used as a positive control. Three duplicate observations were made; bars represent the standard error of the mean.

The time-killing kinetics curve showed that the amount of *L. ivanovii* ATCC19119 increased from 6.63 log_10_CFU/ml to 9.48 log_10_CFU/ml within 10 h in the absence of rEntA. The decrease in the counts of *L. ivanovii* ATCC19119 varied considerably depending on the concentration of rEntA. For example, the maximum viability loss (MVL), which was approximately 0.44 log_10_ CFU/ml (~60% reduction in CFU), was reached within 2 h in 1 × MIC of rEntA. The 2 × MIC of rEntA could cause approximately 1.42 log_10_ CFU/ml viability loss (96% reduction) within 6 h. Moreover, the MVL of *L. ivanovii* treated by rEntA at 4 × MIC was approximately 2.03 log_10_ CFU/ml (>99% reduction in CFU) within 4 h. Although rEntA could inhibit the growth of *L. ivanovii* ATCC19119, the survivors resumed growth at 1× and 2 × MIC of rEntA and 2 × MIC ampicillin for *L. ivanovii* ATCC19119 after MVL was achieved (Figure 3). However, *L. ivanovii* ATCC19119 treated by 4 × MIC of rEntA did not show re-growth within 10 h, revealing that 80 ng/ml rEntA could effectively inhibit the growth of pathogenic bacteria for an extended time.

### Effects of pH, temperature, proteolytic enzymes and NaCl on the activity of rEntA

**Figure 4:**
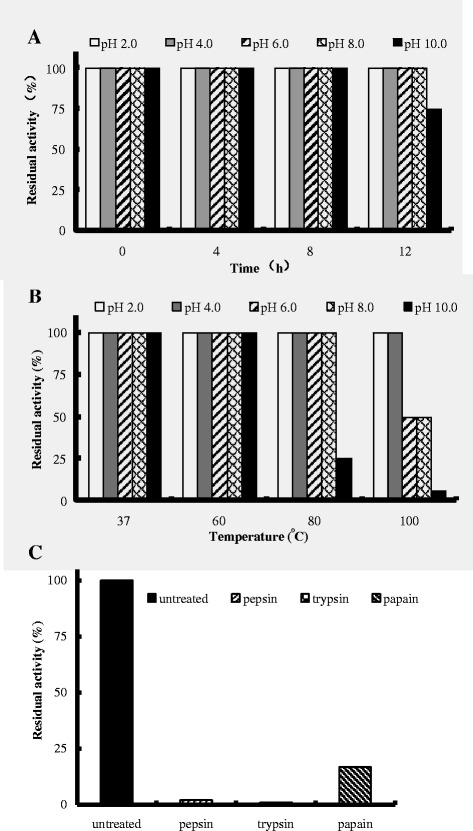
**Effects of pH, temperature and proteolytic enzymes on the rEntA activity. A**, pH stability of rEntA. Purified rEntA was incubated in buffers with a pH range from 2 to 10 at 37°C for 12 h. The initial activity of the sample in a buffer with a pH of 6 was described as 100% activity. **B**, Thermal stability of EntA. Purified rEntA was incubated in buffers with a pH range from 2 to 10 at temperatures of 37, 60, 80, and 100°C for 1 h. The initial activity of the sample in a buffer with a pH of 6 was described as 100% activity. **C**, Proteolysis resistance of rEntA. Purified rEntA was incubated with pepsin, papain and trypsin at 37°C for 4 h. The residual antimicrobial activity of samples was tested after the pH was readjusted to pH 6.0 with sodium phosphate buffer.

As shown in Figure 4A, rEntA was highly stable at a wide range of pH values. The activity of rEntA was maintained completely within a pH range of 2–8 at 37°C for 12 h and was 75% retained even after incubation at a pH of 10 for 12 h. Furthermore, the antimicrobial activity of rEntA was not affected by heat treatment at 37, 60, 80 and 100°C for 1 h under acid conditions (pH 2 and 4) (Figure 4B). The residual activity decreased to 20% at a pH of 10 at 80°C, to 50% at a pH of 6, 8 at 100°C, and to 10% at a pH of 10 at 100°C. In addition, the antimicrobial activity of rEntA was completely abolished by pepsin and trypsin treatment, but it retained 16.7% of initial antimicrobial activity after papain treatment at 37°C for 1 h (Figure 4C).

**Figure 5:**
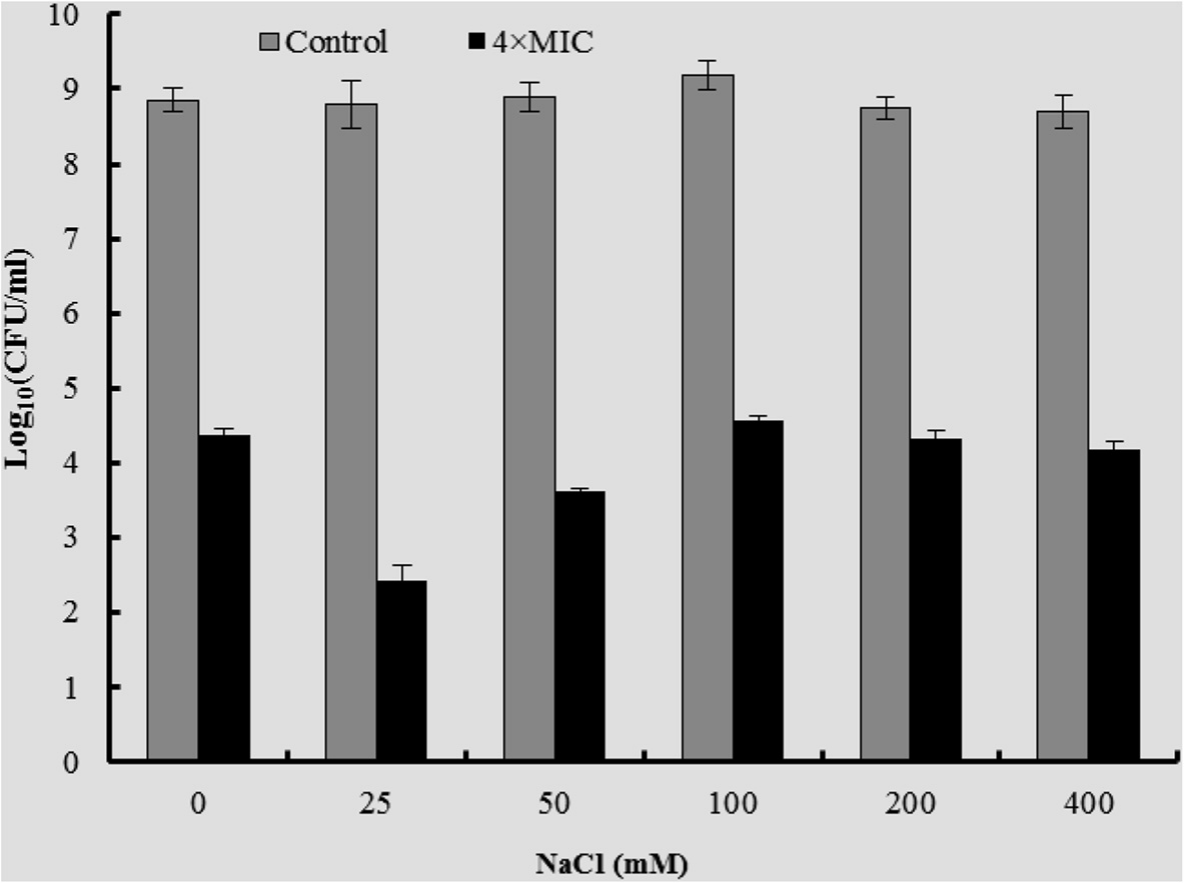
**Effect of NaCl concentration on the activity of rEntA.** Control: *L. ivanovii* ATCC19119 was incubated in the absence of rEntA. 4 × MIC: *L. ivanovii* ATCC19119 was incubated in the presence of rEntA at 4 × MIC.

The antimicrobial activity of rEntA against *L. ivanovii* ATCC19119 was slightly enhanced by the addition of 25 and 50 mM NaCl (Figure 5). The lowest amount of 2.43 log_10_ CFU/ml was observed with a treatment of rEntA (12,800 AU/ml) in 25 mM NaCl (44.52% of that at 0 mM NaCl). The other treatments, from 100 – 400 mM NaCl, had no significant effect on the bactericidal ability of rEntA (Figure 5). In the controls without rEntA, growth was not influenced by NaCl (0 – 400 mM) (Figure 5).

## Discussion

Bacteriocin has attracted attention in recent years for its potential application as a food preservative and therapeutic antimicrobial agent [20]. However, low production of these bacteriocins by native strains cannot meet the requirements of commercial applications. Moreover, some *Enterococci* strains were recognized as opportunistic pathogens associated with lots of infections [21]. Attempts to produce bacteriocins by using safe heterologous hosts have been undertaken in recent years [17,22,23], including some typical expression systems such as *E. coli*, *L. lactis*, and *P. pastoris*. Although *E. coli* and *L. lactis* are widely used in heterologous protein expression because of their easy operation and safety [14,24], they are not suitable for bacteriocins due to toxicity to the host [25] and low recovery percentages from the fusion protein [26]. Many bacteriocins, such as enterocin P [17], hiracin JM79 [27], enterocin L50 [28], pediocin PA-1 [29] and EntA [18], have been expressed as active forms in *P. pastoris*, but their expression levels remained low (below 280 mg/l). It is known that codon optimization is a useful strategy to increase the yield of target protein during heterogeneous expression. Many antimicrobial peptides, such as plectasin [30], NZ2114 [31] and AgPlectasin [32], were expressed with high production through codon-usage optimization in our laboratory. In addition, Divercin V41, a class IIa bacteriocins was also expressed through this system [33]. These cases encouraged us to use codon optimization to break through the bottleneck of low yield in heterologous expression of EntA. The total protein level in the supernatant reached 180 mg/l with the activity of 51,200 AU/ml at 24 h of induction in 5-L fermenter level (Figure 2C) after the gene was optimized. Although the yield of target peptide was still low, and even lower than 280 mg/l as the highest result of expression in case of enterocin L50 in *P. pastoris* [28], it was much higher than that of Pediocin PA-1 (0.4 mg/l), Enterocin P (0.006 mg/l), Divercin V41 (23 mg/l) and EntA (0.027 mg/l) expressed in *E. coli* and *L. lactis* [14,22,33]. Furthermore, the production of rEntA increased 2.99-times compared with its native sequence expressed in *P. pastoris* (45.1 mg/l), which indicated codon optimization is a good tool to enhance expression efficiency and level in *P. pastoris*, and at the same time, it also left a large room to improve in future work at the similar aim and technical scheme.

However, the maximal activity of rEntA in the supernatant was reached at an early stage (24 h) of induction (Figure 2C). This is similar to previous results in which the highest level of rEntA was reached at 36 h. An even earlier peak of rEntA at 6 h was observed in other yeasts such as *Kluyveromyces lactis* and *Hansenula polymorpha* [18]. Obviously, its final successful application suffered from this strong decomposition in the supernatant at an earlier period of expression related to the possible disruption of rEntA to host cells and the proteolysis of the target protein. The latter situation was reported in “collagen-like” bacteriocin with a high cleavage by collagenase [29]. However, the exact mechanism of the above described early degradation and its solution should be further studied.

A series of methods, such as ion exchange chromatography (SP and CM FF), hydrophobic exchange chromatography (Phenyl HP), and gel filtration (Superose 12), were attempted to purify rEntA in this study. Only gel filtration could purify rEntA with a yield of 3.02 mg/l (Figure 2F) after attempts with SP FF, CM FF, and phenyl HP in which almost all rEntA was lost in the penetration peak (data not shown) due to unknown reasons.

Although there are different antibacterial spectrums between class IIa bacteriocins, they consistently have particularly high antibacterial activity against *Listeria* — the most common pathogen in food — at nanomolar concentrations [1]. The MICs of purified native EntA from *E. faecium* T136 against *Listerias* ranged from 40 to 120 ng/ml [34]. Similarly, rEntA also showed a narrow antibacterial spectrum (Table 1) including *L. ivanovii* ATCC19119, and with a low MIC value of 20 ng/ml, it is approximately 20-fold lower than that of ampicillin (390 ng/ml). The re-growth after MVL achievement was a common phenomenon when the *Listeria* was treated with bacteriocins such as EntA, pediocin, sakacin A and enterococcin EFS2 in relatively low concentrations (1× or 2 × MIC) [3], but we found no re-growth after MVL within 10 h when 4 × MIC rEnA was used with the *Listeria* (Figure 3), indicating that higher concentrations of rEnA are essential to inhibit the multiplication of *Listeria*.

The bactericidal activity and overall structure of Pediocin PA-1 and piscicolin 126 were well maintained at higher temperatures [35,36]. The native EntA was stable at 100°C and acidic pH conditions [37]. We found that rEntA also exhibited high stability under a wide range of temperatures (37–80°C) and pH levels (2–8) (Figure 4). These properties were potentially due to the higher cysteine content of the antimicrobial peptides [38], similar to the EntA containing four cysteine residues. In addition, the antimicrobial activity of some bacteriocins (nisin, sakacin P and curvacin A) was significantly enhanced with the addition of NaCl from 0 to 1.17 M [39]. However, the activity of rEntA against *Listeria* was enhanced only at low NaCl concentrations (25 and 50 mM). Despite the unknown mechanisms of the above differential effects, the high stability of rEntA over wide ranges of temperature, pH, and NaCl concentration supports its use as a food preservative and drug candidate.

Due to the high content of basic and aromatic amino acids in class IIa bacteriocins, pediocin PA-1, enterocin B, plantaricin 423 and native EntA were very sensitive to the digestive proteases trypsin and pepsin [11,40,41]. Similarly, the purified rEntA, with 12.76% basic amino acids and 10.63% aromatic amino acids, was inactivated with trypsin and pepsin (Figure 4C). This high sensitivity to digestive proteases of rEntA contributes to its safety in foods and drugs, during and after oral administration.

## Conclusion

In conclusion, rEntA, as an antimicrobial agent with merit, could selectively kill important and pathogenic *Listeria* and retain bio-activity over a wide range of pH values, temperature and NaCl concentrations. These excellent antibacterial properties make it a potential candidate as a food preservative and therapeutic antimicrobial agent. rEntA was successfully expressed in *P. pastoris* X-33 at the highest level of 51,200 AU/ml and was purified through a gel filtration column. This yeast system may be a feasible technological approach to produce rEntA as a potent anti-*Listeria* agent after further optimization.

## Methods

### Strains and vectors

*Escherichia coli* DH5α, *Pichia pastoris* X-33 and pPICZαA were purchased from Invitrogen (Beijing, China). Target strains for the antimicrobial activity assays are listed in Table 2. Restriction enzymes were purchased from New England Biolabs (NEB, Beijing, China). The kits for plasmid extraction and DNA purification were purchased from Tiangen (Beijing, China). Other chemical reagents used in this research were all of analytical grade.

**Table 2:** **Strains used in the antimicrobial activity assays**

**Strains**	**Source**
Gram-positive	
*Listeria ivanovii* ATCC19119	CICC^a^
*Enterococcus faecium* CGMCC1.2136	CGMCC^b^
*Enterococcus faecalis* CGMCC1.130	CGMCC
*Enterococcus faecalis* CGMCC1.2024	CGMCC
*Staphylococcus aureus* ATCC 25923	CVCC^c^
*Staphylococcus epidermidis* ATCC26069	CVCC
*Bacillus licheniformis* CGMCC1.265	CGMCC
*Bacillus coagulans* CGMCC1.2407	CGMCC
*Bacillus subtilis* ATCC6633	CVCC
*Lactococcus lactis*	Stored in our lab
*Bifidobacterium bifidum* CGMCC1.2212	CGMCC
Gram-negative	
*Escherichia. coli* ER2566	CGMCC
*Escherichia. coli* CVCC 195	CVCC
*Escherichia. coli* CMCC 44102	CMCC^d^
*Pseudomonas aeruginosa* CVCC 2087	CVCC
*Salmonella enteritidis* CVCC3377	CVCC

Note: ^a^China Center of Industrial Culture Collection, ^b^China General Microbiological Culture Collection, ^c^China Veterinary Culture Collection, ^d^China Center for Medical Culture Collection.

### Construction of the expression vector and transformation

The optimized EntA gene (GenBank accession No. KJ155693) was generated by the ‘ReverseTranslateTool’ (http://www.bioinformatics.org/sms2/rev_trans.html) according to the codon usage of *P. pastoris* (http://www.kazusa.or.jp/codon/). To express the target protein with a native N-terminus, the Kex2 signal cleavage site was fused to the EntA sequence. The designed sequence was synthesized by Sangon Biotech (Shanghai, China) and digested using *Xho*I and *Xba*I. Resulting DNA fragments were ligated into pPICZαA to generate the recombinant vector pPICZαA-EntA. The latter was transformed into *E. coli* DH5α, and positive transformants were confirmed by DNA sequencing. The recombinant plasmid was linearized with *Pme*I and transformed into *P. pastoris* X-33 competent cells by electroporation [30]. Positive transformants were screened on YPDS medium containing 100 μg/ml of zeocin and further confirmed by colony-PCR.

### Expression of rEntA at the shake-flask level

The positive transformants were grown in BMGY medium until the cultures reached an OD600 nm of 5.0–6.0 at 30°C. Cells were harvested by centrifugation at 4000 rpm for 10 min and resuspended in BMMY medium to an OD600 nm of 1.0. Methanol was added daily to a final concentration of approximately 0.5%. Samples were taken at 0, 12, 24, 36, 48, 60 and 72 h for analysis.

### Expression of rEntA at the fermenter level

A single colony of *P. pastoris* X-33 (pPICZαA-EntA) was grown in 10 ml of YPD medium at 30°C overnight. The culture was inoculated into 200 ml fresh YPD medium and cultivated at 29°C to an OD600 nm of approximately 6.0. The 200-ml seed culture was transferred into a 5-L fermenter (Sartorius StedimBiotech) containing 1.8 L of basal salt medium with 45 g/L of NH_4_H_2_PO_4_, 20 g/L K_2_SO_4_, 0.4 g/L CaSO_4_, 15 g/L MgSO_4_ 7H_2_O, 6 g/L KH_2_PO_4_, 1.5 g/L KOH, and 200 ml 45% w/v glucose. The initial fermentation was a glucose batch phase (approximately 18 h). After exhaustion of the glucose, 50% w/v glucose was added for 6 h at a feed rate of 36 ml/h. After the glucose was exhausted, methanol was supplied from 2 to 12 ml/h. The whole fermentation period was performed at 29°C. During the glucose batch and glucose-fed phases, the pH was kept at 5.0 and increased to 5.5 at the methanol induction phase [42]. The protein in the supernatant was determined by the Bradford protein assay (Tiangen, Beijing, China) and Tricine-SDS–PAGE [43].

### Purification of rEntA

The supernatant with rEntA from *P. pastoris* X-33 (pPICZαA-EntA) X-33 was desalted by a gel filtration column (Sephadex G-25) with a flow rate of 2 ml/min and then freeze-dried and dissolved in 100 mM of ammonium acetate buffer. The sample was passed through a gel filtration column (Superose 12) and eluted with the same buffer at a flow rate of 0.5 ml/min. Purified rEntA was further lyophilized to remove ammonium acetate.

### Antimicrobial activity assay

Tested strains including *L. ivanovii*, *E. faecalis*, and *E. faecium* were grown in Mueller-Hinton (MH) broth containing 3% fetal bovine serum (FBS). *S. epidermidis*, *B. subtilis*, *L. lactis*, *B. bifidum*, *B. licheniformis*, *B. coagulans* and *S. aureus* were grown in MH broth. *P. aeruginosa*, *E. coli* and *S. enteritidis* were grown in LB medium. All tested strains were grown to 0.4 of OD600 nm at 37°C. One hundred microliters of the cell suspension was inoculated into 50 ml of preheated medium containing 1.5% agar. This was rapidly mixed and poured into a Petri dish. Sterile Oxford cups were put on the surface of the solidified media. Each cup was filled with 50 μl of samples [30].

Titer assays were used to quantify the antimicrobial activity of rEntA according to the method of Liu [12]. The titer was expressed as arbitrary units (AU/ml). One arbitrary unit (AU) was defined as the reciprocal of the highest dilution showing a clear zone of inhibition to the indicator strain. When a clear inhibition zone was followed by a turbid one, the critical dilution was taken to be the average of the final two dilutions.

Minimal inhibitory concentrations (MICs) and Minimum bactericidal concentrations (MBCs) assays were determined using the microtiter broth dilution method [30]. Ampicillin was also tested with the same concentration gradient as a positive control. All tests were performed in triplicate.

### In-vitro killing curve assay

To evaluate the antibacterial activity of rEntA against *L. ivanovii* ATCC19119, a time-kill assay was performed as described by the methods of Mao [32]. In addition, tubes with only bacterial inoculum were used as growth controls. All experiments were performed in triplicate.

### Effects of pH, temperature, proteases and NaCl on the activity of rEntA

The effects of pH, temperature and proteases on rEntA activity were determined as described previously [30,44] with the following modifications: 1) The titer of purified rEntA used in the test was 12,800 AU/ml; 2) The initial activity of sample in the buffer with a pH of 6 was taken as 100% activity for pH and thermal stability assays; and 3) The residual antimicrobial activity of samples was tested after the pH was readjusted to 6.0 with sodium phosphate buffer (pH 6.0) for the proteolytic sensibility assay.

To evaluate the effect of NaCl concentration on the activity of rEntA, an overnight culture of *L. ivanovii* ATCC19119 was diluted to 10^5–6^ CFU/ml in fresh MHB medium (3% FBS). Ten microliters of purified rEntA and 10 μl of NaCl solution were added to 80 μl of diluted cell culture. The final rEntA concentration was 4 × MIC, and the final NaCl concentrations were 0, 25, 50, 100, 200, and 400 mM. Samples without rEntA were used as controls. All samples were incubated at 37°C for 10 h. The CFU of tested strains was determined. All tests were performed in triplicate.
